# TBK1-stabilized ZNF268a recruits SETD4 to methylate TBK1 for efficient interferon signaling

**DOI:** 10.1016/j.jbc.2023.105428

**Published:** 2023-11-04

**Authors:** Yi Liu, Wei Yin, Xianhuang Zeng, Jinhao Fan, Chaozhi Liu, Mingyu Gao, Zan Huang, Guihong Sun, Mingxiong Guo

**Affiliations:** 1 Hubei Key Laboratory of Cell Homeostasis, College of Life Sciences, Wuhan University, Wuhan, Hubei, P.R. China; 2 Taikang Medical School (School of Basic Medical Sciences), Wuhan University, Wuhan, Hubei, P.R. China; 3 School of Ecology and Environment, Key Laboratory of Biodiversity and Environment on the Qinghai-Tibetan Plateau of Ministry of Education, Tibet University, Lhasa, Tibet, P.R. China; 4 Hubei Provincial Key Laboratory of Allergy and Immunology, Wuhan, Hubei, P.R. China

**Keywords:** species-specific regulation of innate immunity, ZNF268a, TBK1 methylation, SETD4, virus-induced interferon signaling

## Abstract

Sufficient activation of interferon signaling is critical for the host to fight against invading viruses, in which post-translational modifications have been demonstrated to play a pivotal role. Here, we demonstrate that the human KRAB-zinc finger protein ZNF268a is essential for virus-induced interferon signaling. We find that cytoplasmic ZNF268a is constantly degraded by lysosome and thus remains low expressed in resting cell cytoplasm. Upon viral infection, TBK1 interacts with cytosolic ZNF268a to catalyze the phosphorylation of Serine 178 of ZNF268a, which prevents the degradation of ZNF268a, resulting in the stabilization and accumulation of ZNF268a in the cytoplasm. Furthermore, we provide evidence that stabilized ZNF268a recruits the lysine methyltransferase SETD4 to TBK1 to induce the mono-methylation of TBK1 on lysine 607, which is critical for the assembly of the TBK1 signaling complex. Notably, ZNF268 S178 is conserved among higher primates but absent in rodents. Meanwhile, rodent TBK1 607th aa happens to be replaced by arginine, possibly indicating a species-specific role of ZNF268a in regulating TBK1 during evolution. These findings reveal novel functions of ZNF268a and SETD4 in regulating antiviral interferon signaling.

The antiviral innate immune response is elicited swiftly upon the detection of invading viral nucleic acid through pathogen-associated molecular patterns (PAMPs) (1). Several RNA and DNA sensors, including RIG-I, MDA5, cGAS, and IFI16, have been identified for detecting cytosolic viral genomes (2). Following the detection of viral nucleic acid, signaling is propagated *via* either mitochondria-located MAVS (3) (also named IPS-1 (4), VISA (5), or Cardif (6)) or endoplasmic reticulum-located STING (7) (also named ERIS (8), MITA (9), or MPYS (10)). The activation of MAVS or STING recruits Tank-binding kinase 1 (TBK1) and interferon regulatory factor 3 (IRF3), thus triggering phosphorylation-dependent activation and nuclear translocation of IRF3 (11), resulting in the transcription of interferon β (IFNβ) and other IFN-stimulated genes (ISGs) (12), which establishes an antiviral state of host cells and further promotes the induction of adaptive immunity (13).

During the activation of interferon, TBK1 is an essential component and lies in the center of the IRF3 signaling cascade (14). The activation of TBK1 is tightly regulated *via* various post-translational modifications (PTMs) (15). It has been reported that multiple PTMs, including phosphorylation (16), ubiquitination (17, 18, 19), and acetylation (20, 21), positively or negatively regulate TBK1 activity. Most recently, arginine methylation of TBK1 was also reported to promote TBK1 activation, further expanding our knowledge of TBK1 regulation (22). However, it remains unknown whether other non-canonical PTMs, such as lysine methylation, are involved in regulating TBK1 activity. Therefore, it would be interesting to explore the role of lysine methylation in regulating interferon signaling.

KRAB-zinc finger proteins constitute the largest family of tetrapod-specific transcription factors (23). At present, studies on KRAB-zinc finger protein family members are mainly focused on their functions as transcription factors in the fields of biological evolution, embryonic development, and cancer (24, 25), and the total number of studies is minimal compared to the large gene number (23, 25). In other areas of biology, little is known about whether KRAB-zinc finger proteins have functions other than acting as transcription factors.

Species-specific regulation has brought increasing attention to understanding human innate immunity (14, 26). Traditionally, the mouse is widely used as a model animal in investigating innate immune systems. A large number of milestone discoveries are found using mouse models (27). Despite the importance of mouse models, growing evidence has shown that the human innate immune system differs in many ways from the mouse system (28, 29). For example, Burleigh *et al.* (28) reported that though both human and mouse primary fibroblasts and primary MEF cell lines could respond to DNA transfection characterized by STING degradation, HSPA8 phosphorylation could only be activated in the human fibroblasts. In addition, Jin and colleagues showed that type Ⅰ interferon production was elevated in human Tetherin KO cells but repressed in plasmacytoid dendritic cells in Tetherin KO mice (29).

ZNF268a is primarily a nucleus-resident protein and is long considered to be a transcriptional regulator (30). In this study, we report a novel role of ZNF268a in facilitating antiviral innate immune signaling in the cytoplasm. We find that ZNF268a can specifically target the critical kinase TBK1 and help maintain the interaction between TBK1 and MAVS/STING to facilitate the activation of IRF3. Mechanistically, cytoplasmic ZNF268a can be degraded by the Tollip-mediated selective autophagy system in the absence of viral challenge. However, when viral infection occurs, TBK1 interacts with cytosolic ZNF268a and directly phosphorylates Serine 178 of ZNF268a, which could promote the protein stability of ZNF268a and significantly increase the expression level of ZNF268a in the cytoplasm. With the stabilization of ZNF268a, it can recruit lysine methyltransferase SETD4 to TBK1, which directly induces the mono-methylation of lysine 607 in TBK1, thus maximizing TBK1 activation. Unexpectedly, we demonstrate that ZNF268a Serine 178 and TBK1 lysine 607 are conserved residues across primates but are absent or highly variable in rodents, likely suggesting a species-specific regulation of innate immunity in human beings.

## Results

### ZNF268a is required for the interferon response to RNA and DNA viruses

**Figure 1:**
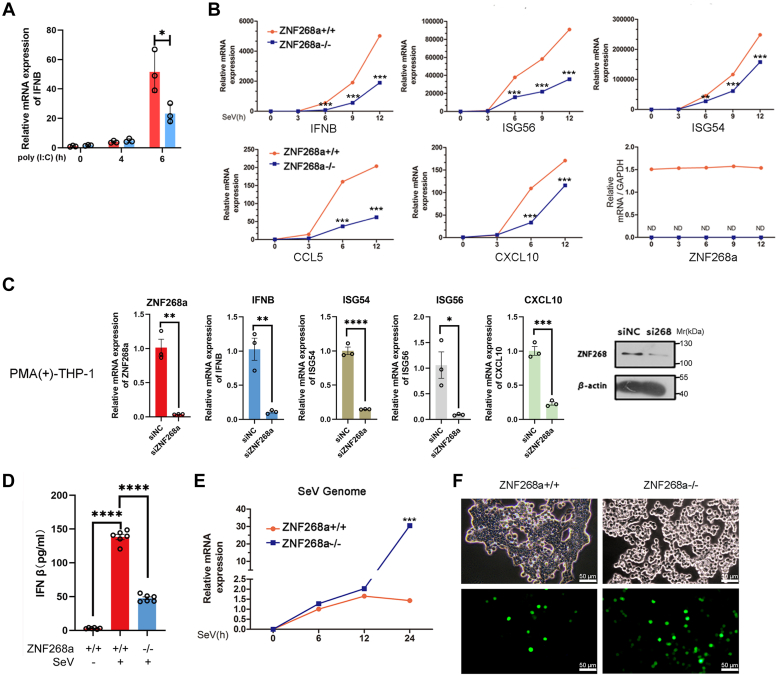
**ZNF268a is required for the interferon response to RNA and DNA viruses.***A*, qPCR analysis of IFNB mRNA in ZNF268a+/+ and ZNF268a−/− HEK293T cells for indicated time points with transfected poly(I:C). *B*, qPCR analysis of IFNB, ISG56, ISG54, CCL5, CXCL10, and ZNF268a mRNA in ZNF268a+/+ and ZNF268a−/− HEK293T cells infected for various times with SeV. *C*, PMA-differentiated ZNF268a knock-down THP-1 cells were treated with HSV-1 for 12 h, with IFNB, ISG54, ISG56, CXCL10, and ZNF268a mRNA expression in siRNA NC-treated cells set at 100%. *D*, quantification by ELISA of secreted IFN-β in the supernatant of ZNF268a+/+ and ZNF268a−/− HEK293T cells after SeV infection for 12 h. *E*, qPCR analysis of SeV genome RNA in ZNF268a+/+ and ZNF268a−/− HEK293T cells at indicated time points. *F*, wide-field fluorescent microscopy of GFP-VSV infection in ZNF268a+/+ and ZNF268a−/− HEK293T cells. Scale bar, 50 μm. Values are expressed as the mean ± SEM and unpaired two-tailed Student’s t-tests are used for statistical analysis in which ∗*p* < 0.05, ∗∗*p* < 0.01, ∗∗∗*p* < 0.001, ∗∗∗∗*p* < 0.0001. Data are representative of three independent experiments.

**Supplemental Fig. S1:**
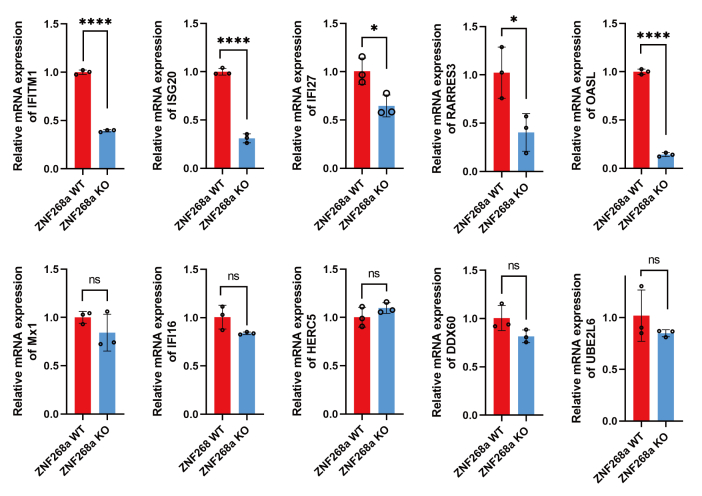
**Related to**Figure 1**ISGs affected and unaffected by ZNF268a deficiency during viral infection.** ZNF268a wild-type and knockout cells were infected by SeV for 12 h and the indicated ISGs were quantified by qRT-PCR. Values are expressed as the mean ± SEM and unpaired two-tailed Student’s t-tests are used for statistical analysis in which ∗*p* < 0.05, ∗∗∗∗*p* < 0.0001 and ns means not significant. Data are representative of two independent experiments.

Previously, we demonstrated a positive role of ZNF268a in regulating virus-induced proinflammatory cytokines (31). To further investigate whether ZNF268a is essential in facilitating effective host interferon response against viral infection, we first used our previously established ZNF268a KO HEK293T cell line (31) to test the effect of ZNF268a on interferon response induced by poly(I:C), which is a synthetic analog of double-stranded RNA (dsRNA) and commonly used as a molecular pattern associated with viral infections-induced interferon transcription. Lack of ZNF268a attenuated IFN-β transcription 6 h post poly(I:C) transfection (Fig. 1*A*). Next, we stimulated the cells with SeV for 12 h and measured the transcripts of IFN-β and ISGs of the cultured cells by quantitative real-time PCR. Knock-out of ZNF268a largely abrogated IFN-β, ISG54, ISG56, CXCL10, and CCL5 transcription induced by SeV in HEK293T cells during the time points indicated in Figure 1*B*. In addition, we tested other ISGs including IFITM1, ISG20, IFI27, RARRES3, OASL, MX1, IFI16, HERC5, DDX60, and UBE2L6, among which the expression of MX1, IFI16, HERC5, DDX60 and UBE2L6 is not affected by ZNF268a deficiency (Fig. S1). Furthermore, down-regulation of ZNF268a in PMA-induced THP-1 cells caused up to 75% lower expression of IFN-β, ISG54, ISG56, and CXCL10 in response to DNA virus HSV-1 infection (Fig. 1*C*). Consistent with the qPCR data, ELISA results showed that ZNF268a-deficient HEK293T cells produced significantly less IFN-β in response to SeV infection (Fig. 1*D*). Accordingly, SeV genome copies were much higher in ZNF268a KO HEK293T than in WT cells (Fig. 1*E*). In addition, the deletion of ZNF268a impaired HEK293T cells’ resistance to GFP-VSV infection, as shown by stronger GFP intensity in ZNF268a KO cells by microscopy (Fig. 1*F*). Collectively, these data indicated a positive role for ZNF268a in regulating the RNA/DNA viral infection-induced interferon response.

### Virus-induced IRF3 signaling is impaired by ZNF268a deficiency

**Figure 2:**
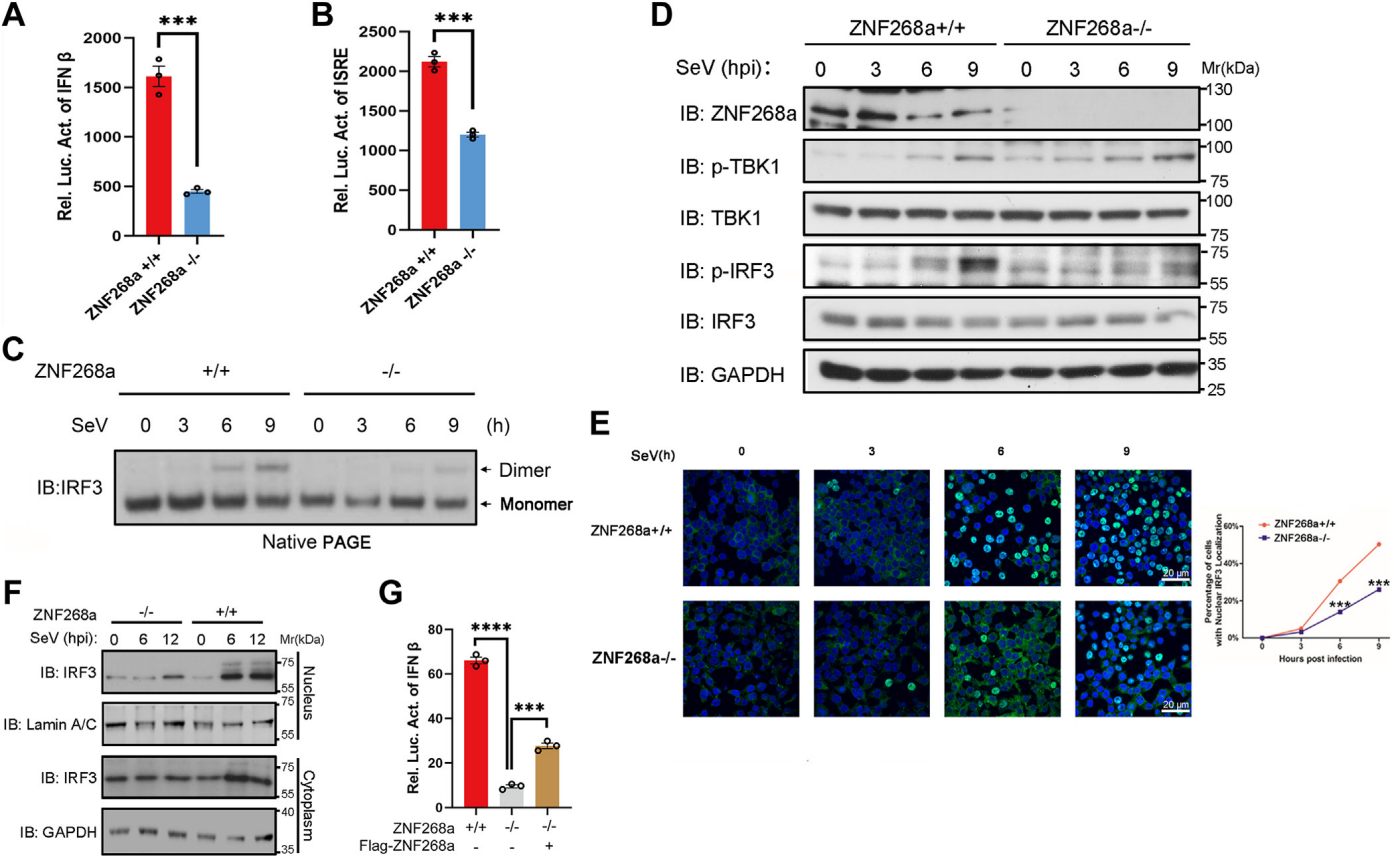
**Virus-induced IRF3 signaling is impaired by ZNF268a deficiency.***A*, wild-type and ZNF268a-knockout cells were transfected with IFN-β luciferase reporter plasmids. Luciferase activity was measured after treatment with SeV for 12 h. *B*, wild-type and ZNF268a-knockout cells were transfected with ISRE luciferase reporter plasmids. Luciferase activity was measured as in (*A*). *C*, IB of IRF3 dimerization in ZNF268a+/+ and ZNF268a−/− HEK293T cells infected with SeV for the indicated hours. *D*, IB of p-IRF3, p-TBK1, total IRF3 and TBK1 in ZNF268a+/+ and ZNF268a−/− HEK293T cells infected with SeV for indicated time points. *E*, fluorescent images and percentage quantification of IRF3 translocation in wild-type and ZNF268-knockout HEK293T cells treated with SeV for the indicated time points. Scale bar, 20 μm. *F*, IB of the nuclear and the cytoplasmic IRF3 in ZNF268a+/+ and ZNF268a−/− HEK293T cells infected with SeV for the indicated time points. *G*, IFN-β promoter luciferase assay of wild-type, ZNF268-knockout HEK293T cells and Flag-ZNF268a transfected ZNF268-knockout HEK293T cells treated with SeV for 12 h. Values are expressed as the mean ± SEM and unpaired two-tailed Student’s t-tests are used for statistical analysis in which ∗∗∗*p* < 0.001. Data are representative of three independent experiments.

**Supplemental Fig. S2:**
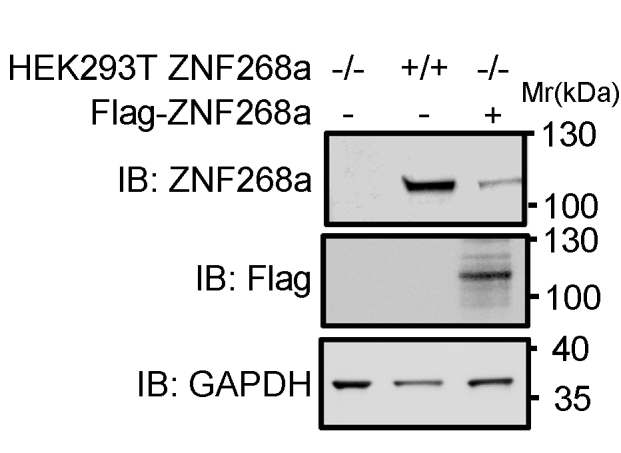
**Related to**Figure 2**insufficient expression efficiency of ZNF268a construct.** Flag-ZNF268a was introduced into ZNF268a−/− HEK293T cells. The expression of the construct is confirmed by the Flag antibody blot; the expression of ZNF268a in the WT and the ZNF268a-rescued KO cells were blotted by the antibody against the endogenous ZNF268a. Data are representative of two independent experiments.

To better understand how ZNF268a regulates the innate immune responses to viral infection, we compared the cell response to SeV infection in WT or ZNF268a KO HEK293T cells by performing IFN-β/ISRE promoter dual luciferase assay. As shown in Figure 2, *A* and *B*, ZNF268a deficiency significantly reduced the activation of both promoters, indicating that the deletion of ZNF268a impaired the virus-induced interferon signaling pathway. On the other hand, we observed decreased activation of IRF3 upon SeV infection in ZNF268a KO HEK293T cells, as revealed by native PAGE that IRF3 dimerization was decreased in ZNF268a KO HEK293T cells (Fig. 2*C*), which corresponded to the attenuated level of phospho-S396 IRF3 when ZNF268a was depleted (Fig. 2*D*). Following viral infection, IRF3 nuclear translocation was also repressed in ZNF268a deficient HEK293T cells (Fig. 2*E*). In line with the imaging data, the biochemical fractionation assay also indicated that the level of nuclear IRF3 accumulated stronger and faster in ZNF268a wild-type cells than in ZNF268a knockout cells (Fig. 2*F*). However, notably, we did not observe a concurrent decrease in TBK1 phosphorylation of S172 (Fig. 2*D*), suggesting that the phosphorylation-dependent activation of TBK1 was largely unaffected by ZNF268a depletion. Furthermore, re-introduction of the full-length construct of ZNF268a partially restored IFN-β promoter activation upon SeV infection (Fig. 2*G*). Poor expression level could be the cause for the partial restore (Fig. S2), as we noticed similar examples in other KRAB-ZNF proteins such as ZFP809, which was reported to express poorly in differentiated HEK293A but regularly in undifferentiated embryonic stem cells (32, 33). In general, the result demonstrated that the impaired IRF3 signaling activation was due to the specific loss of ZNF268a.

### ZNF268a targets TBK1 to facilitate IRF3 activation

**Figure 3:**
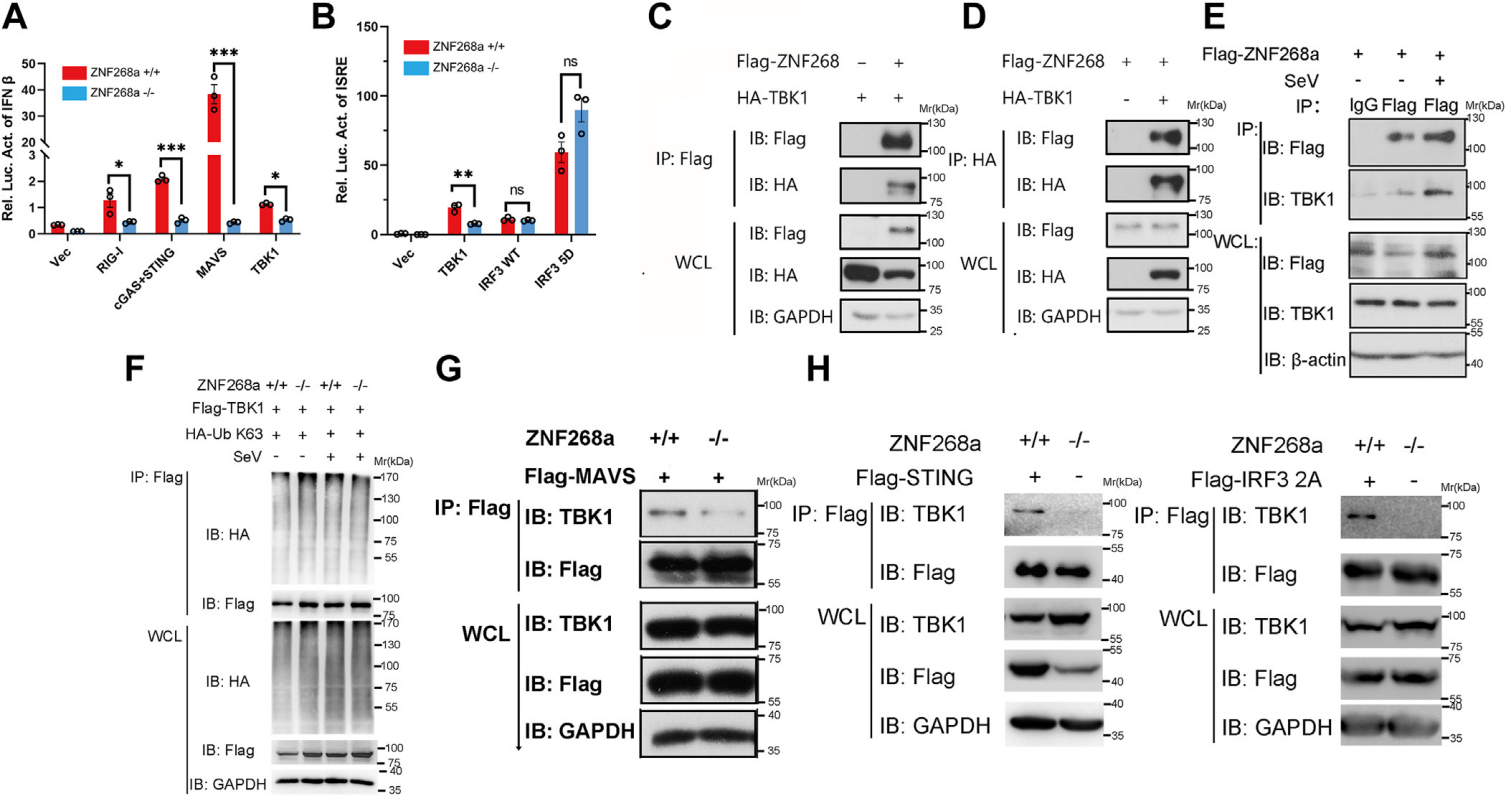
**ZNF268a targets TBK1 to facilitate IRF3 activation.***A* and *B*, luciferase reporter assays revealed that ZNF268a KO inhibited various signaling factors overexpression-induced IFN-β (*A*) and ISRE (*B*) promoter transactivation. *C* and *D*, lysates from HEK293T cells transfected with the indicated plasmids for 24 h were subjected to immunoprecipitation with anti-Flag (*C*) or HA (*D*) antibodies followed by Western blot analysis with anti-HA or Flag antibodies. *E*, lysates from HEK293T cells transfected with Flag-ZNF268a plasmids for 24 h were subjected to immunoprecipitation with anti-Flag antibody and immunoblotted with endogenous TBK1 antibody. *F*, IB of whole cell lysates and immunoprecipitants derived from ZNF268a WT and KO HEK293T cells transfected with K63-linked HA-Ub plasmids with Flag-TBK1, treated with or without SeV for 8 h. *G* and *H*, Endogenous TBK1 of whole cell lysates and immunoprecipitants derived from ZNF268a WT and KO HEK293T cells transfected with Flag-MAVS (*G*), STING (*H*, *left*) and IRF3-2A (*H*, *right*) plasmids. Values are expressed as the mean ± SEM and unpaired two-tailed Student’s t-tests for statistical analysis in which ∗*p* < 0.05, ∗∗*p* < 0.01, ∗∗∗*p* < 0.001, and ns means not significant. Data are representative of three independent experiments.

To further identify which component of the interferon signaling pathway is targeted by ZNF268a, we overexpressed RIG-I, cGAS + STING, MAVS, TBK1, IRF3 WT or its constitutively active form IRF3 5D constructs individually, in HEK293T cells. We found that ZNF268a deficiency inhibited RIG-I, cGAS + STING, MAVS, and TBK1 overexpression-induced activation of IFN-β promoters (Fig. 3*A*) but had little effect on IRF3 WT or IRF3 5D-induced activation of ISRE promoter (Fig. 3*B*), suggesting that ZNF268a could potentially target TBK1 to affect interferon signaling. To confirm the role of ZNF268a at the level of TBK1, we examined the interaction of Flag-tagged ZNF268a with HA-tagged TBK1. Immunoprecipitation assay using either Flag (Fig. 3*C*) or HA (Fig. 3*D*) antibody demonstrated that Flag-ZNF268a was associated with HA-TBK1. Due to the lack of suitable ZNF268a antibody for immunoprecipitation, we exogenously expressed Flag-tagged ZNF268a in cells and used Flag antibody to perform immunoprecipitation and immunoblotted for endogenous TBK1. The result showed that Flag-ZNF268a was indeed able to bind endogenous TBK1 (Fig. 3*E*), further supporting the association of ZNF268a and TBK1.

Since ZNF268a can associate with TBK1 without affecting TBK1 phosphorylation, we speculated that the attenuated interferon responses observed in ZNF268a KO HEK293T cells could be due to impaired assembly of the TBK1 signaling complex or decreased Lys63-linked ubiquitination since it is reported to be critical for TBK1 activation (17, 34). Detection of TBK1 Lys63-linked ubiquitination in ZNF268a WT and KO HEK293T cells with or without viral infection showed no significant difference, excluding the possibility of ZNF268a in regulating TBK1 activation *via* K63 ubiquitination (Fig. 3*F*). In contrast, co-immunoprecipitation assays showed a robustly diminished association between TBK1 and MAVS, STING or IRF3 in ZNF268a KO HEK293T cells (Fig. 3, *G* and *H*). Thus, we concluded that TBK1 is the target of ZNF268a in regulating virus-induced activation of IRF3 signaling.

### Viral infection prevents lysosomal degradation of ZNF268a *via* TBK1

**Figure 4:**
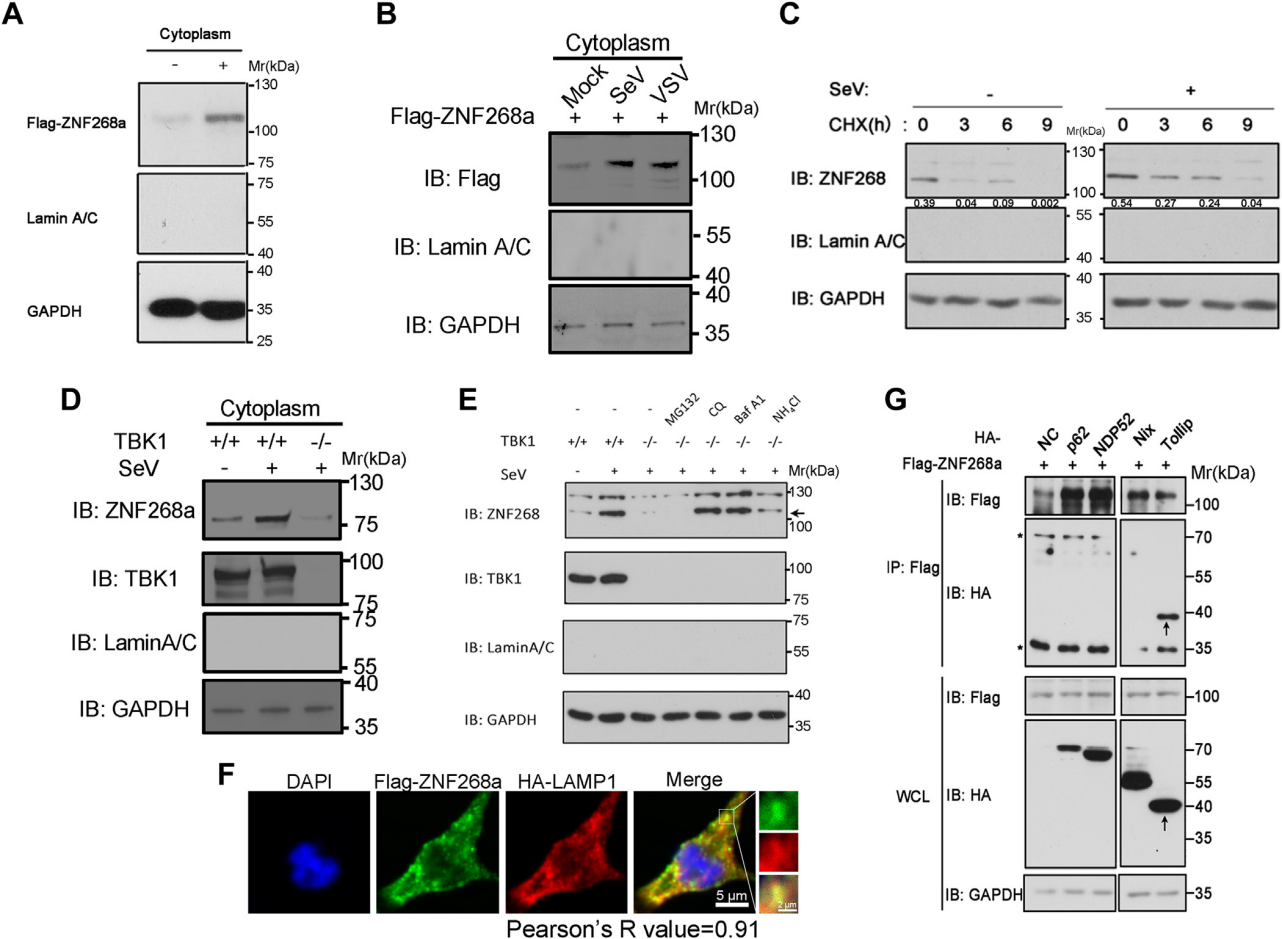
**Viral infection prevents lysosomal degradation of ZNF268a *via* TBK1.***A*, SeV-infected HEK293T cells were subjected to subcellular fractionation, and cytoplasmic Flag-ZNF268a expression was probed by immunoblot. *B*, cytoplasmic Flag-ZNF268a expression was probed as in (*A*), while both SeV and VSV were used to infect cells. *C*, the degradation rate of cytoplasmic ZNF268a from HEK293T cells treated with or without SeV, measured by CHX chase assay. *D*, IB of endogenous cytoplasmic ZNF268a in parental and TBK1 knockout HEK293T cells infected with SeV for 12 h. The arrow denotes specific bands. *E*, IB of endogenous cytoplasmic ZNF268a in HEK293T cells infected with SeV for 8 h. Cells were left untreated or treated with chloroquine (CQ) (100 mM), Baflomycin A1 (0.2 μM), NH_4_Cl (20 mM), and MG132 (10 μM), for 6 h before harvest. The arrow denotes specific bands. *F*, confocal microscopy of HEK293T cells transfected with Flag-ZNF268a and HA-LAMP1. Scale bar, 5 μm. Scale bar of the inset, 2 μm. *G*, HEK293T cells were transfected with plasmids encoding Flag-ZNF268a and Flag-tagged key proteins in selective autophagy (HA-p62, HA-NDP52, HA-Nix, HA-Tollip), followed by IP with anti-Flag antibody and immunoblot analysis with anti-HA. The *arrow* denotes specific bands and the *asterisk* denotes non-specific bands. Data are representative of three independent experiments.

As shown in Figure 1*B*, the transcriptional level of ZNF268a remains unaltered during viral infection. We further evaluated if its protein level changed during viral infection. We transfected Flag-tagged ZNF268a into HEK293T cells and challenged the cells with SeV. We found a drastically elevated protein expression of cytoplasmic ZNF268a in the infected cells (Fig. 4*A*). Similar results were repeatedly obtained in VSV-infected HEK293T cells (Fig. 4*B*). We also measured the degradation rate of cytoplasmic ZNF268a using the CHX chase assay. The result showed that viral infection substantially extended the half-life of cytoplasmic ZNF268a (Fig. 4*C*). The fact that TBK1 physically interacts with ZNF268a leads us to hypothesize that TBK1-ZNF268a interaction could be critical for the virus-induced stabilization of ZNF268a. To examine this hypothesis, we analyzed the ZNF268a protein level in HEK293T cells with a deficiency in TBK1. Deletion of TBK1 blocked virus-induced cytoplasmic ZNF268a protein elevation (Fig. 4*D*). The cytoplasmic upregulation of ZNF268a raised the possibility that exposure to the viral nucleic acid can release ZNF268a from its constant degradation by cell quality control systems, which mainly consist of the ubiquitin-proteasome system and the autolysosome system. To further determine the mechanism by which the ZNF268a protein level is increased after infection, we treated cells with proteasome or lysosome inhibitors. As the result in Figure 4*E* showed, lysosome degradation inhibition by chloroquine, Baflomycin A1, or NH_4_Cl restored the ZNF268a protein expression, whereas proteasome degradation inhibition by MG132 did not. Colocalization of ZNF268a with lysosome marker LAMP1 further confirmed cytoplasmic ZNF268a is constantly targeted for degradation in lysosome (Fig. 4*F*). Moreover, we found that ZNF268a could specifically associated with selective autophagic receptor Tollip (Fig. 4*G*), suggesting a tight control of the protein level of ZNF268a in resting cells and potentially explaining why transient transfected ZNF268a construct expresses poorly in cells. Together, these data show that viral infection can prevent lysosomal degradation of ZNF268a in a TBK1-dependent manner.

### TBK1 phosphorylates ZNF268a at S178 to stabilize ZNF268a protein expression

**Figure 5:**
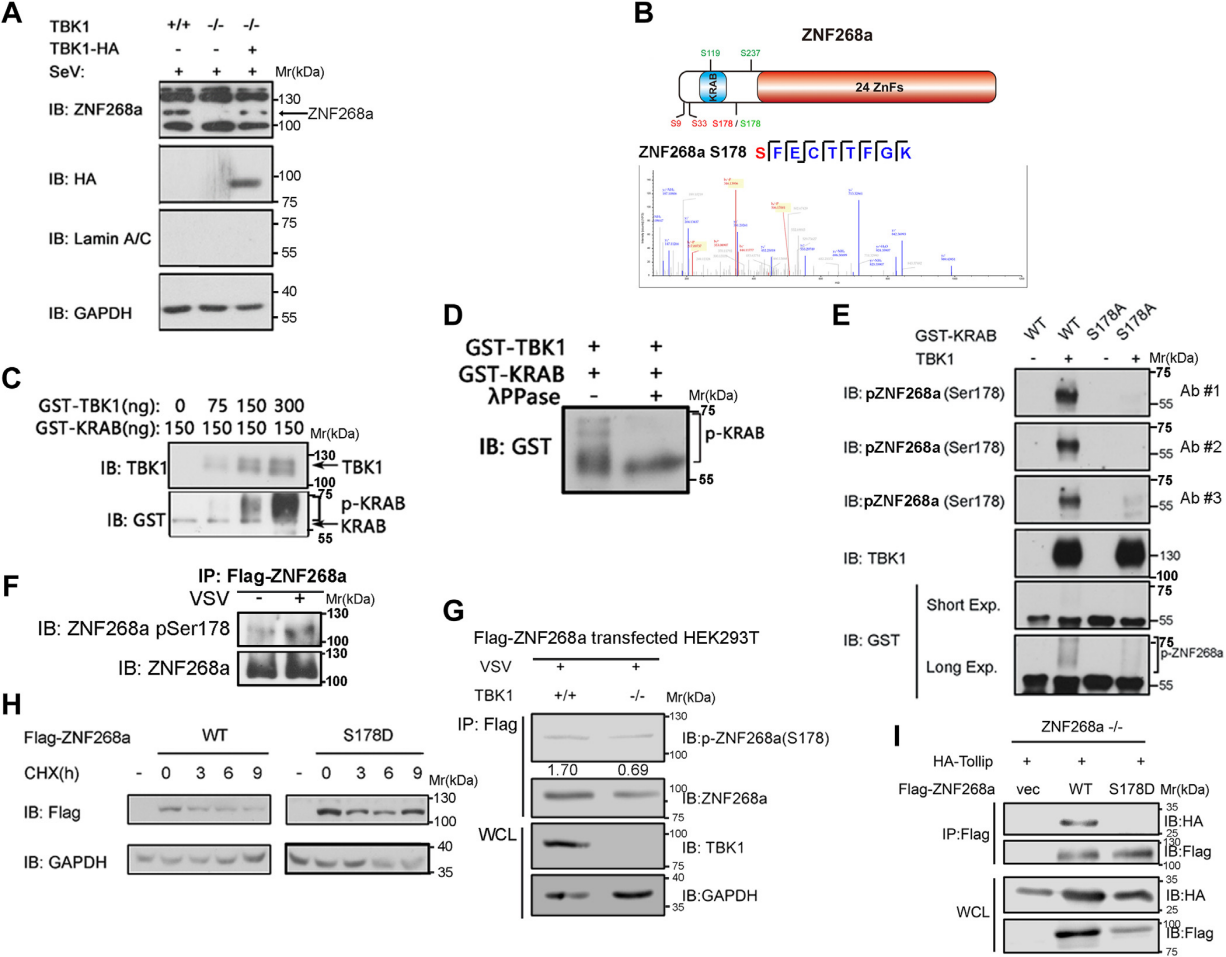
**TBK1 phosphorylates ZNF268a at S178 to stabilize ZNF268a protein expression.***A*, HA-TBK1 was re-introduced to TBK1 KO HEK293T cells and endogenous cytoplasmic ZNF268a was detected with SeV infection for 8 h. *Arrow* denotes specific bands. *B*, *upper*: schematic diagram of TBK1-triggered ZNF268a phosphorylation sites, predicted by bioinformatic analysis (*green*) and identified by MS (*red*). *Lower*: Mass spectrometry identification of ZNF268a Ser178 phosphorylation. *C*, *in vitro* phosphorylation assay using an increasing amount of TBK1 and recombinant ZNF268a N-terminus (referred to as GST-KRAB). *D*, IB of *in vitro* phosphorylation assay product treated with λPPase. *E*, IB of *in vitro* phosphorylation assay using customized pS178 antibodies. *F*, IB of anti-pS178 and anti-ZNF268a immunoprecipitants from HEK293T cells treated with VSV for 8 h. *G*, IB of anti-pS178 and anti-ZNF268a immunoprecipitants from TBK1 WT and KO HEK293T cells treated with VSV for 8 h. *H*, Degradation rate of cytoplasmic Flag-ZNF268a WT and S178D construct from ZNF268a KO HEK293T cells, measured by CHX chase assay. *I*, Co-IP and immunoblot analysis of 293T cells transfected with HA-Tollip and Flag-ZNF268a WT, S178D and empty vector. Data are representative of three independent experiments.

In order to further understand how TBK1 mediates ZNF268a protein stabilization, we restored TBK1 WT expression in TBK1 KO HEK293T cells and found an accumulation of cytoplasmic ZNF268a, confirming the critical role of TBK1 in stabilizing cytoplasmic ZNF268a (Fig. 5*A*). Since TBK1 is a well-known serine/threonine kinase, we speculated that the interaction of TBK1 and ZNF268a could lead to the phosphorylation of ZNF268a, further resulting in its stabilization. Bioinformatic analysis predicted three potential phospho-sites located in the N-terminus of ZNF268a, including S119, S237, and S178 (Fig. 5*B* upper, sites in green). We next performed mass spectrometry analysis of Flag-tagged ZNF268a in the presence or absence of viral infection (Fig. 5*B* lower). The analysis identified Ser9, Ser33, and Ser178 as specific phosphorylation sites (Fig. 5*B* upper, sites in red). *In vitro* kinase assay using recombinant TBK1 and the N-terminus of ZNF268a (in which the whole KRAB domain and surrounded sequences were included while all ZnF motifs were excluded, so we termed this truncated protein as GST-KRAB for convenience) showed that increasing amount of TBK1 triggered stronger smear bands of ZNF268a N-terminus (Fig. 5*C*), which was not detected upon incubation of the reaction products with λ-phosphatase (Fig. 5*D*), indicating TBK1 was able to phosphorylate ZNF268a at its N-terminus.

As both the bioinformatic prediction and the MS result identified Ser178 phosphorylation of ZNF268a (Fig. 5*B* upper), we focused on this amino acid in our following experiments. To confirm that TBK1 phosphorylates ZNF268a at Ser178, we generated three independent antibodies recognizing Ser178-phosphorylated ZNF268a. Immunoblot analysis with these antibodies showed that TBK1 efficiently phosphorylated ZNF268a N-terminus WT at Ser178 *in vitro*, whereas phosphorylation of ZNF268a N-terminus S178A was not detected (Fig. 5*E*). Notably, in HEK293T cells, VSV infection induced Ser178 phosphorylation of immunoprecipitated Flag-ZNF268a (Fig. 5*F*), whereas loss of TBK1 abrogated this effect (Fig. 5*G*).

To confirm the importance of ZNF268a Ser178 phosphorylation in stabilizing ZNF268a, we performed a CHX chase assay to determine the degradation rate of ZNF268 WT and S178D phospho-mimetic mutant. As expected, ZNF268a S178D degraded slower than its WT counterpart (Fig. 5*H*). Of note, when ZNF268a Serine 178 was mutated to aspartic acid, the interaction of ZNF268a and Tollip was almost abolished (Fig. 5*I*), implying that ZNF268a S178 phosphorylation-mediated stabilization was likely *via* diminishing the interaction with Tollip, thus preventing the targeted degradation.

To further investigate the effect of ZNF268a Ser178 phosphorylation on antiviral innate immunity, we compared IRF3 dimerization in ZNF268a WT, S178D, and S178A overexpressed ZNF268a KO HEK293T cells. SeV infection could induce IRF3 dimerization in WT and S178D rescued cells, but this dimerization was compromised in S178A-expressed cells (Fig. S5*A*). In line with the effects of IRF3 dimerization, IFN-β transcription was restored in WT ZNF268a transfected cells, but this restoration was utterly lost in S178A expressing cells (Fig. S5B). These data suggested that ZNF268a S178 phosphorylation plays an essential role in the antiviral interferon signaling transduction.

### ZNF268a is required for the recruitment of methyltransferase SETD4 to TBK1

**Supplemental Fig. S4:**
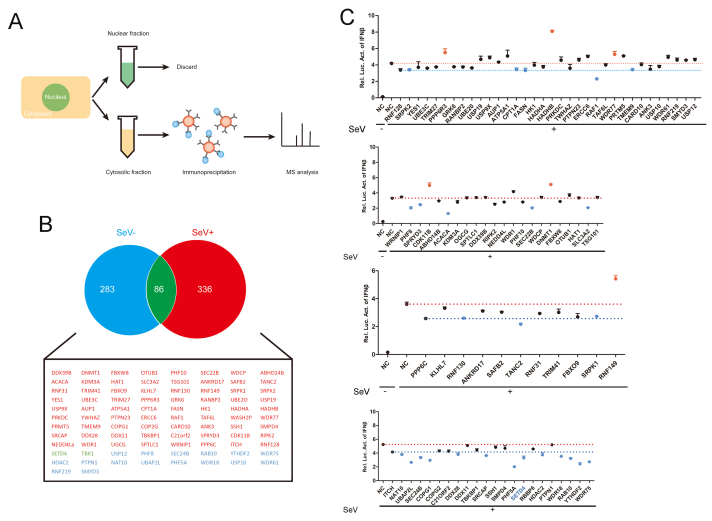
**Related to**Figure 6**identification and validation of ZNF268a binding partners in interferon signaling regulation.***A*, schematic diagram of cytoplasmic ZNF268a interacting protein identification. *B*, mass spectrometry analysis of cytoplasmic ZNF268a interacting proteins with or without SeV infection for 8 h. *C*, HEK293T cells were transfected by a pool of 3 siRNAs per target, IFN-β reporter assays were performed to identify virus-induced interferon response regulators. Values are expressed as the mean ± SEM and unpaired two-tailed Student’s t-tests are used for statistical analysis in which ∗*p* < 0.05, ∗∗*p* < 0.01, ∗∗∗*p* < 0.001, ∗∗∗∗*p* < 0.0001. Data are representative of two independent experiments.

To systemically elucidate how ZNF268a regulates the TBK1 signaling complex, we performed mass spectrometry to identify potential ZNF268a interacting partners during viral infection. As depicted in Fig. S4A, we subjected Flag-tagged ZNF268a overexpressed cytoplasmic fraction but not whole cell lysate to immunoprecipitation to avoid potential interference of nuclear binding partners of ZNF268a, followed by MS identification of ZNF268a interacting proteins. *Via* this approach, we identified 422 ZNF268a interacting proteins when cells were infected by SeV (Fig. S4B). Among these protein candidates, we chose 90 to further verify their potential roles in antiviral innate immunity based on our interest in post-translational modifications. We tested these proteins by knocking down their expression *via* a pool of three individual siRNAs per target in a luciferase reporter assay (Fig. S4C). Notably, downregulation of SETD4, the only identified methyltransferase, attenuated the activation of IFN-β reporter following SeV infection (Fig. S4C, the lowest panel).

**Figure 6:**
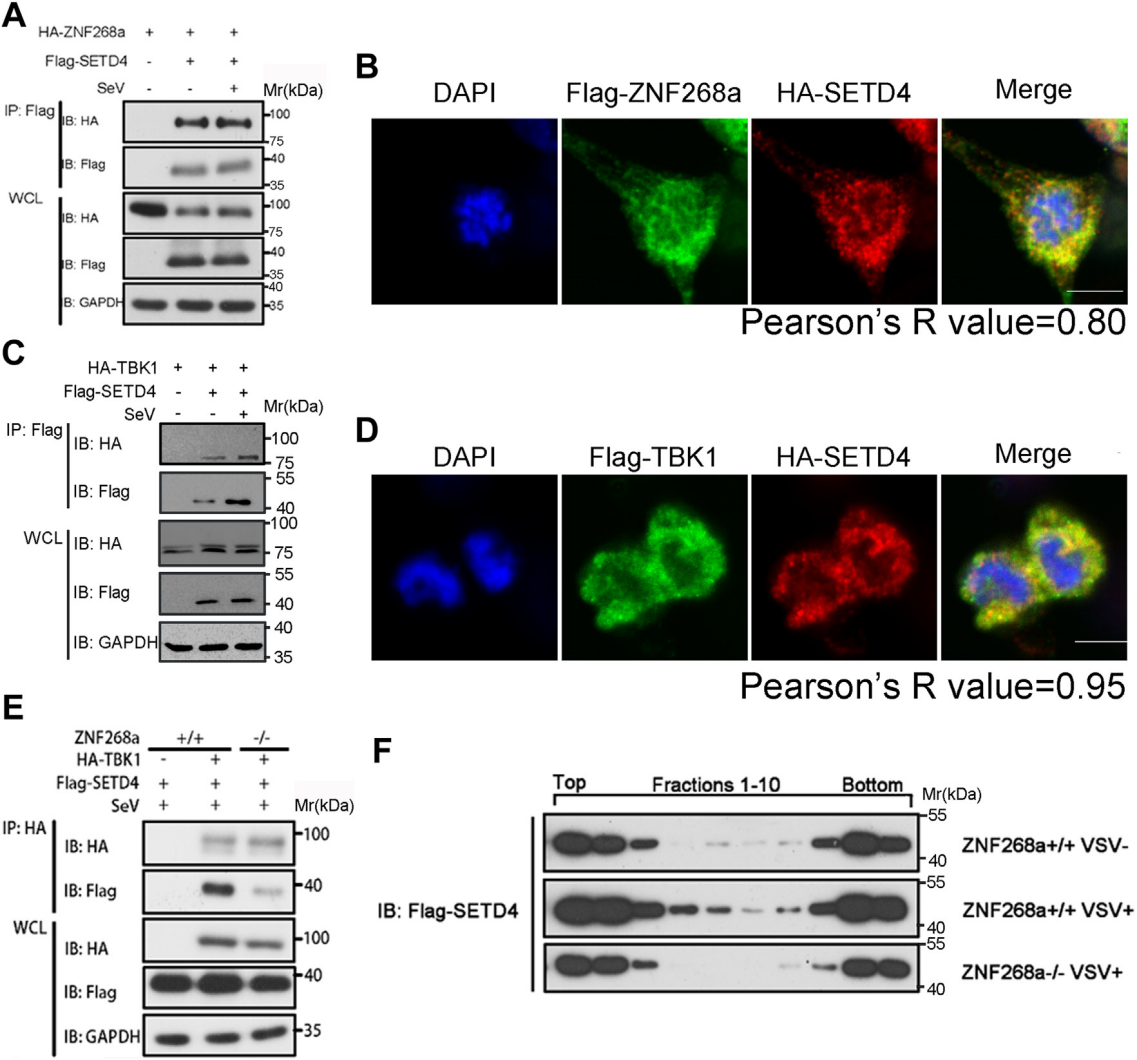
**ZNF268a recruits methyltransferase SETD4 to TBK1.***A*, Co-IP and immunoblot analysis of 293T cells transfected with HA-ZNF268a and Flag-SETD4 with or without SeV infection for 8 h. *B*, Representative fluorescent images of Flag-ZNF268a and HA-SETD4 in HEK293T cells (N = 8). Scale bar: 10 μm. *C*, Co-IP and immunoblot analysis of 293T cells transfected with HA-TBK1 and Flag-SETD4 with or without SeV infection for 8 h. *D*, representative fluorescent images of Flag-TBK1 and HA-SETD4 in HEK293T cells (N = 12). Scale bar: 10 μm. *E*, Co-IP and immunoblot analysis of ZNF268a+/+ and ZNF268a−/− HEK293T cells transfected with HA-TBK1 and Flag-SETD4 with SeV infection for 8 h. *F*, cytoplasmic fractions isolated from Flag-SETD4 overexpressed ZNF268a+/+ and ZNF268a−/− HEK293T cells infected with or without VSV for 8 h were subjected to sucrose gradient ultracentrifugation. Aliquots of the fractions were immunoblotted with Flag antibody. Data are representative of three independent experiments.

Then, we constructed HA-tagged SETD4 and performed a Co-IP experiment to further verify its interaction with ZNF268a. Our result suggested that overexpressed SETD4 was sufficient to associate with ZNF268a even at resting state (Fig. 6*A*). Immunofluorescence analysis also confirmed the co-localization of exogenously expressed ZNF268a and SETD4 in the cytoplasm (Fig. 6*B*). Importantly, we detected clear interaction between SETD4 and TBK1 *via* Co-IP both in the uninfected and the SeV-infected HEK293T cells (Fig. 6*C*). IF assay also supported the conclusion that SETD4 bound TBK1 by demonstrating that they co-localized in cells (Fig. 6*D*). Furthermore, we also found SETD4 interacted with TBK1 in a ZNF268a-dependent manner, as ZNF268a depletion strongly impaired this interaction (Fig. 6*E*). Interestingly, analysis of SETD4 protein complex by sucrose gradient ultracentrifugation showed that the Flag-tagged SETD4 in the lysate of non-infected cells, mainly sediments as either a higher-order complex or a lower-order complex (Fig. 6*F*). Virus infection strongly shifted SETD4 from the lightest or heaviest fractions to the middle-molecular weight fractions, also in a ZNF268a-dependent manner (Fig. 6*F*). These results suggested that the SETD4’s behaviors, including TBK1 association and incorporation into higher order molecular complex, were critically dependent on ZNF268a. Together, we concluded that ZNF268a mediates the recruitment of SETD4 to TBK1 and associated complexes upon viral infection.

### SETD4 is essential for antiviral innate immunity

**Figure 7:**
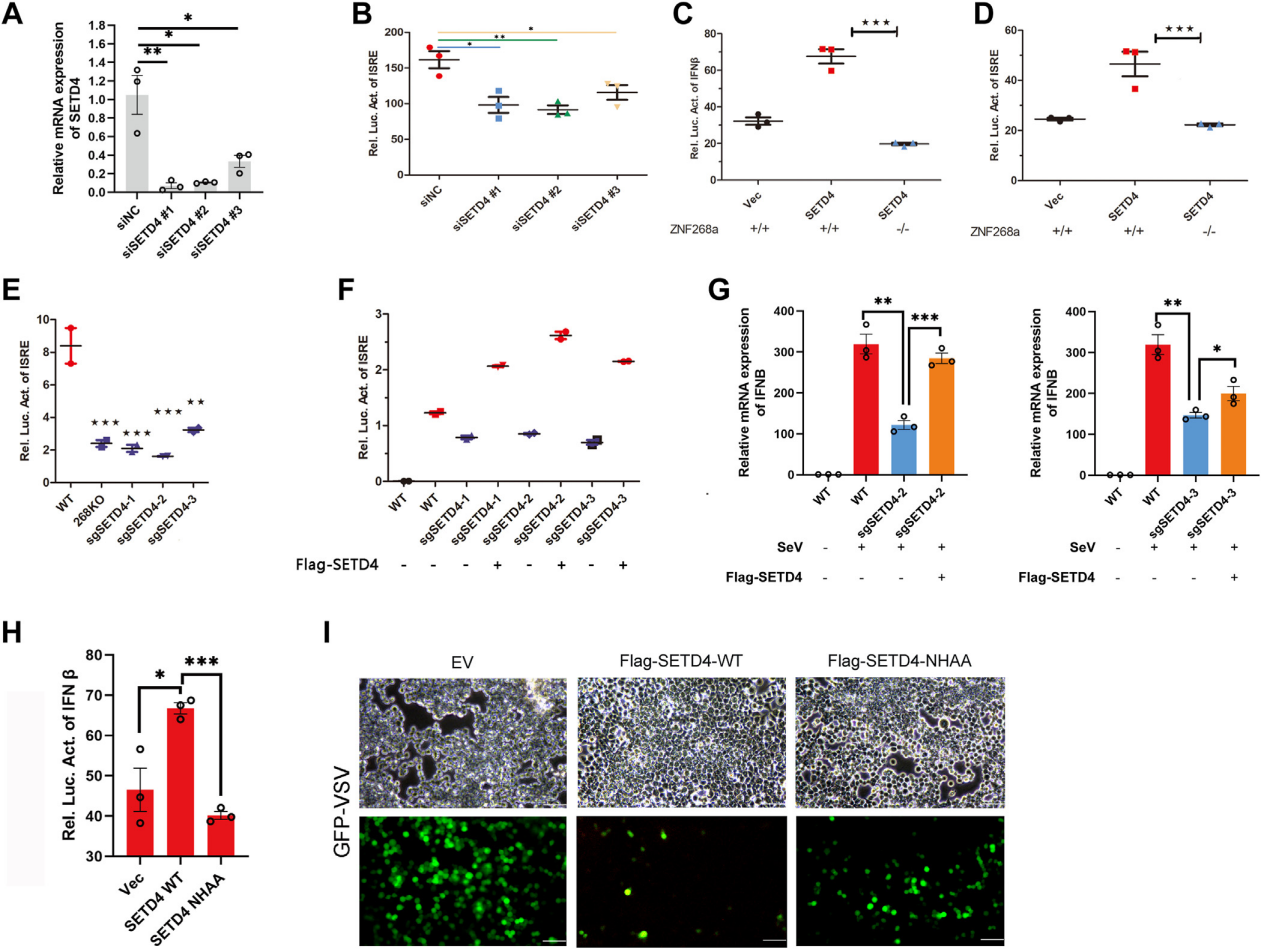
**SETD4 is essential for antiviral innate immunity.***A*, qPCR analysis of efficiency of three siRNAs-mediated STED4 knockdown. *B*, ISRE luciferase reporter assay of siNC- and siSETD4-treated HEK293T cells infected with SeV for 12 h. *C* and *D*, Wild-type and ZNF268a-knockout cells were transfected with Flag-Vector and SETD4 along with IFN-β (*C*) or ISRE (*D*) luciferase reporter plasmids. Luciferase activity was measured after treatment with SeV for 12 h. *E*, sgRNAs against SETD4 were transfected into HEK29T cells and antibiotic-selected cell pools were used to perform luciferase reporter assay as in (*B*). *F*, ISRE luciferase reporter assay of re-introduction of SETD4 into the sgRNA-treated cell pools in (*E*). *G*, qPCR analysis of IFNB of the sgRNA-treated cell pools treated as in (*F*). *H*, IFN-β luciferase reporter assay of HEK293T transfected with empty vector, Flag-SETD4 WT, and kinase-dead NHAA mutant. *I*, Bright-field microscopy (*top*) and fluorescence microscopy (*bottom*) of GFP-VSV in HEK293T cells transfected with empty vector, Flag-SETD4 WT, and kinase-dead NHAA mutant, infected with GFP-VSV for 12 h. Scale bars, 100 μm. Values are expressed as the mean ± SEM and unpaired two-tailed Student’s t-tests are used for statistical analysis in which ∗*p* < 0.05, ∗∗*p* < 0.01, ∗∗∗*p* < 0.001. Data are representative of three independent experiments.

To further examine the function of SETD4 in antiviral innate immunity, we knocked down SETD4 *via* siRNAs in HEK293T cells (Fig. 7*A*) and measured ISRE reporter activation by viral infection. siRNA-mediated knockdown of SETD4 resulted in drastically decreased activation of the ISRE promoter (Fig. 7*B*). In ZNF268a WT HEK293T cells, overexpression of SETD4 could potentiate virus-induced activation of IFN-β/ISRE promoters, which were abolished in ZNF268a KO HEK293T cells (Fig. 7, *C* and *D*), suggesting that ZNF268a was required for STED4-mediated interferon response. Next, we transfected three sgRNAs individually into HEK293T cells and tested the response of these cells in the ISRE dual luciferase assay. Similar to what we observed in siRNA-treated cells, virus-induced activation of ISRE promoter was all inhibited significantly in sgRNA-transfected cells (Fig. 7*E*). Re-introduction of SETD4 could rescue the activation defect (Fig. 7*F*). Finally, as expected, we observed similar results in IFN-β transcription level *via* qRT-PCR (Fig. 7*G*).

Interestingly, we found that WT SETD4 construct potentiated SeV-induced IFN-β promoter activation (Fig. 7*H*). By homology alignment and previous publication (35, 36), we created a predicted methyltransferase-deficient mutant of SETD4 (N286A/H287A, we used NHAA mutant for short). The SETD4 NHAA mutant lost the ability to promote SeV-induced IFN-β promoter activation (Fig. 7*H*). In line with this result, overexpression of WT but not mutant SETD4 repressed GFP-VSV propagation in HEK293T cells (Fig. 7*I*). Therefore, these results implied an essential role of SETD4 methyltransferase activity in facilitating antiviral innate immunity.

### Mono-methylation of TBK1 by SETD4 is critical for interferon response

**Figure 8:**
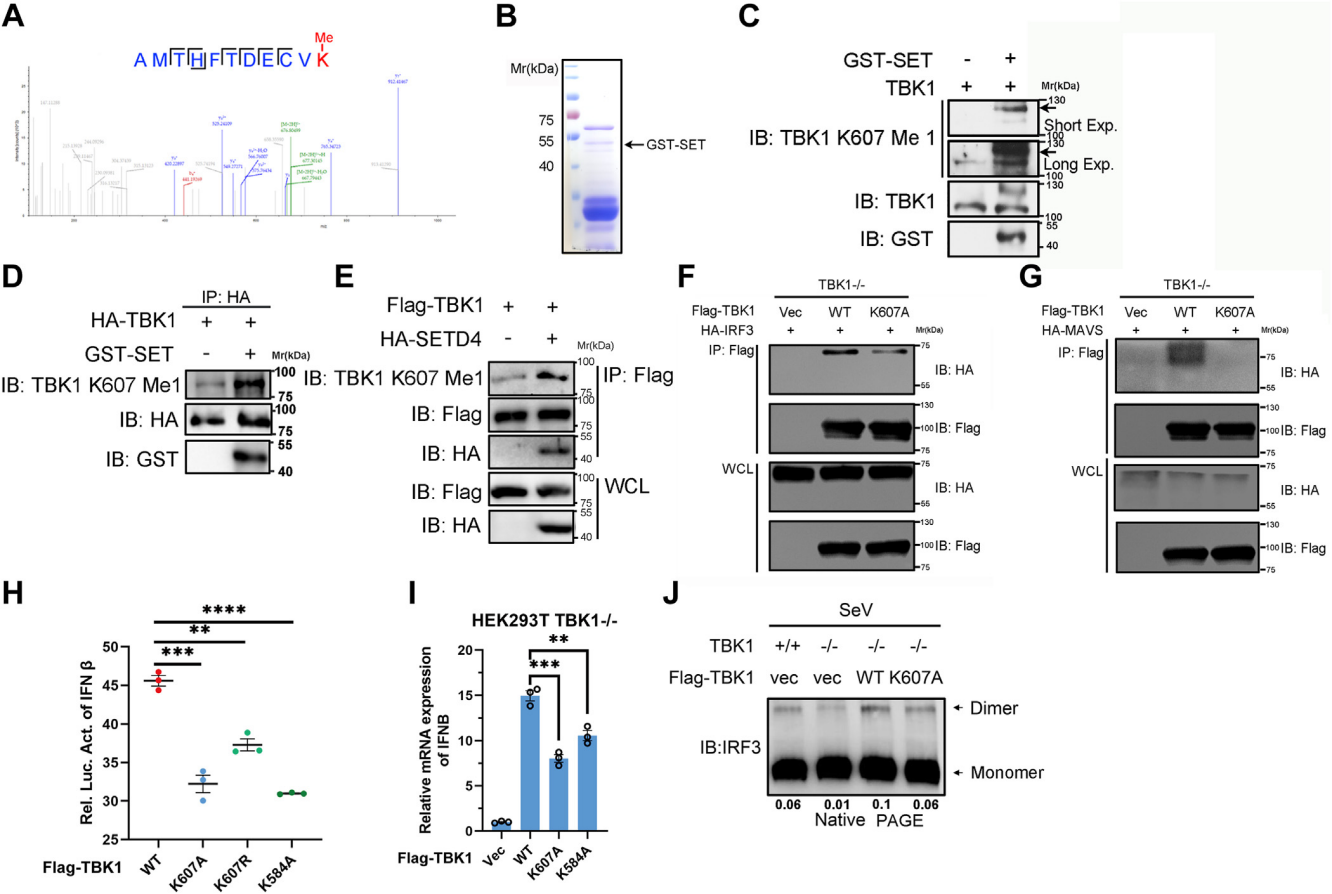
**Mono-methylation of TBK1 by SETD4 is critical for interferon response.***A*, mass spectrometry analysis identified TBK1 K607 mono-methylation site triggered by SETD4 in *in vitro* methylation assay. *B*, Coomassie brilliant blue staining of GST-tagged SETD4 SET domain fusion protein. *C*, IB of TBK1 K607 mono-methylation induced by recombinant SET domain of SETD4 using customized K607 Me specific antibody in an *in vitro* assay. *D*, IB of TBK1 K607 mono-methylation using affinity purified HA-TBK1 from HEK293T as substrate *in vitro*. *E*, Flag-TBK1 was transfected into HEK293T with or without HA-SETD4, followed by anti-Flag immunoprecipitation and IB for TBK1 K607 mono-methylation. *F* and *G*, Co-IP and immunoblot analysis of TBK1 KO HEK293T cells transfected with Flag-TBK1 WT and K607A mutant, along with HA-IRF3 (*F*) or HA-MAVS (*G*) with SeV infection for 8 h. *H*, TBK1 KO HEK293T cells were transfected with IFN-β reporter plasmid and expression plasmids for TBK1 WT, K607A, K607R and K584A. The luciferase activity was then analyzed. *I*, TBK1 KO HEK293T cells were transfected with expression constructs for TBK1 WT, K607A, K584A and infected with SeV for 12 h. IFNB transcription was measured by qPCR. *J*, TBK1 WT and K607A constructs were re-introduced into TBK1 KO HEK293T cells, IRF3 dimerization was detected by native PAGE and immunoblot. Values are expressed as the mean ± SEM and unpaired two-tailed Student’s t-tests are used for statistical analysis in which ∗∗*p* < 0.01, ∗∗∗*p* < 0.001. Data are representative of three independent experiments.

**Supplemental Fig. S5:**
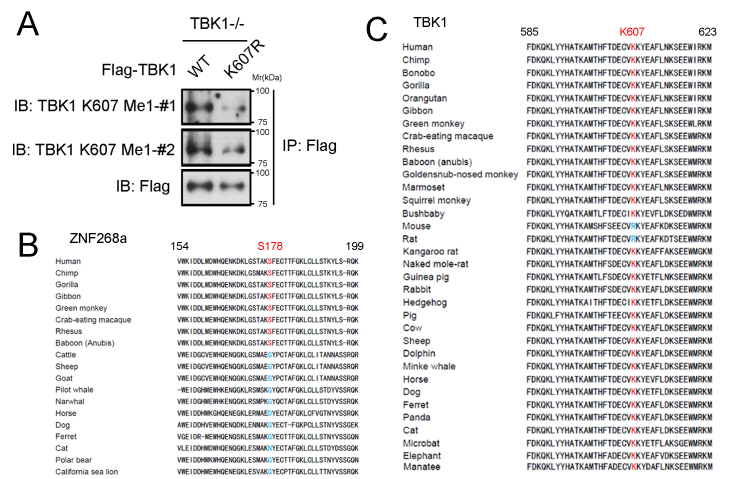
**Conservation of ZNF268a S178 and TBK1 K607 in various species.***A*, antibody specificity validation by IB detecting immunoprecipitated Flag-TBK1 WT and K607R from TBK1 KO HEK293T cells. *B*, alignment of ZNF268a Ser178 flanking sequences of different species. *C*, Alignment of TBK1 K607 flanking sequences of different species.

Based on SETD4-TBK1 interaction and SETD4 is predicted to be methyltransferase, we next investigated whether SETD4 could catalyze TBK1 methylation. By incubating Flag-tagged SETD4 purified from HEK293T cells with recombinant TBK1, followed by MS analysis, we identified the only mono-methylation site of Lys607 on TBK1 (Fig. 8*A*). To verify this result, we generated two antibodies specific to K607-monomethylated TBK1. We tested their specificity by detecting immunoprecipitated Flag-TBK1 WT and K607R mono-methylation from HEK293T cells. The result showed that these antibodies could recognize TBK1 K607 mono-methylation, though with some extent of non-specific signal (Fig. S5A). To verify TBK1 K607 mono-methylation *in vitro*, we expressed and purified the SET domain of SETD4 fused with an N-terminal GST tag (Fig. 8*B*). *In vitro* methylation assay was then performed by incubating the *E. coli*-expressed SET domain of SETD4 with commercially purchased TBK1. Immunoblot analysis revealed that the recombinant SET domain was able to induce mono-methylation of K607 on TBK1 protein (Fig. 8*C*). To further understand SETD4-mediated TBK1 K607 mono-methylation, we transiently transfected HEK293T cells with HA-TBK1 construct and affinity-purified TBK1 protein using anti-HA magnetic beads. As shown in Figure 8*D*, under the above condition, the recombinant SET domain of SETD4 could enhance mono-methylation of immunoprecipitated HA-TBK1. To test whether SETD4 indeed methylated TBK1 K607 in cells, we co-transfected HA-STED4 and Flag-TBK1 constructs into HEK293T, followed by Flag antibody immunoprecipitation. As expected, we found that TBK1 K607 mono-methylation was significantly increased in SETD4-overexpressed cells (Fig. 8*E*). This was consistent with our *in vitro* methylation assay results, further strengthening the conclusion that SETD4 could catalyze TBK1 mono-methylation on Lys607.

To verify the biological function of K607 mono-methylation, we tested whether K607 mono-methylation could affect TBK1 signaling complex assembly in cells. Using co-immunoprecipitation, we observed decreased binding between TBK1 K607A and IRF3 (Fig. 8*F*) or MAVS (Fig. 8*G*) in TBK1 KO HEK293T cells.

In accordance with these results, TBK1 K607A/R mutant expressed in TBK1 KO HEK293T cells showed less IFN-β promoter activation, close to a positive control of TBK1 K584A mutant (37) (Fig. 8*H*). We also observed a similar result in IFN-β transcription (Fig. 8*I*). Furthermore, TBK1 K607 mutant-expressed TBK1 KO HEK293T cells also exhibited attenuated dimerization of IRF3 compared to WT TBK1-expressed cells (Fig. 8*J*). These results suggested that TBK1 K607 mono-methylation was critically associated with virus-induced interferon response.

Interestingly, mice and rats do not have a direct ortholog to human ZNF268 (Fig. S5B). Accordingly, an arginine coincidently replaces the lysine at position 607 of TBK1 in mice and rats (Fig. S5C), possibly suggesting a species-specific regulation of TBK1 K607 methylation in higher primates rather than in model animals like mice and rats.

## Discussion

**Figure 9:**
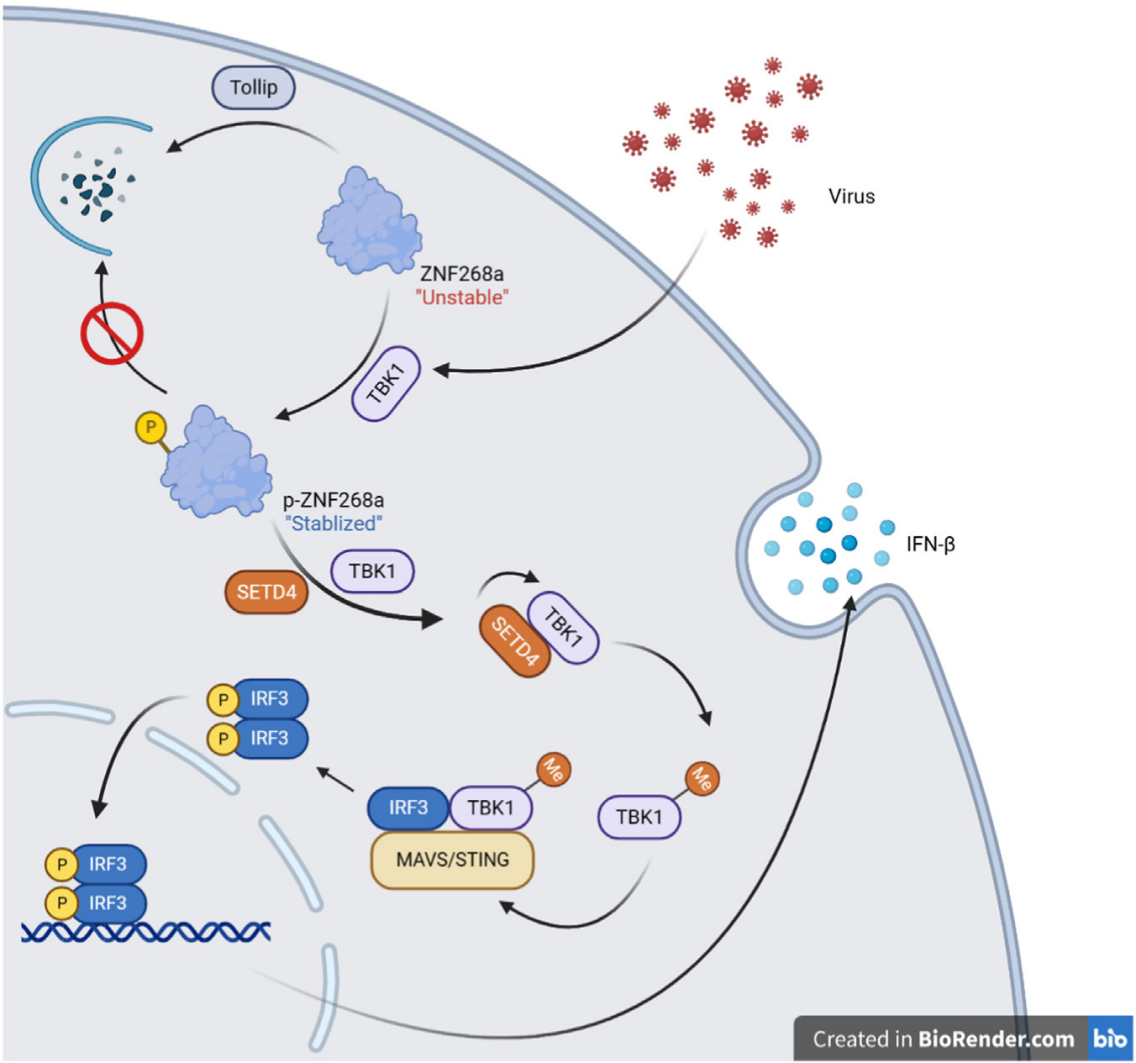
**Working model of ZNF268a in regulating virus-induced interferon signaling.** In uninfected cells, cytoplasmic ZNF268a is targeted by selective autophagic receptor Tollip and subjected to constant degradation *via* autolysosome. When cells are challenged with viral infection, TBK1-mediated S178 phosphorylation of ZNF268a helps maintain protein stability, thus preventing the degradation of ZNF268a. Stabilized ZNF268a, in turn, recruits SETD4 to TBK1 to mono-methylate TBK1 K607, resulting in enhanced TBK1 interaction with MAVS, STING and IRF3, which ultimately promotes interferon production to combat invading viral infection.

The innate immune response is the first line of defense against viral infection. A rapid and efficient activation of innate immune signaling is of vital importance for hosts to combat invading viruses. Post-translational modification provides an effective strategy to regulate signaling activation. Here, we demonstrate that the human KRAB-zinc finger protein ZNF268a is required for the virus-induced interferon signaling *via* mediating TBK1 mono-methylation. Cytoplasmic ZNF268a is constantly degraded by autophagosomes. Thus, the expression remains low in the cytoplasm. Upon viral infection, TBK1 interacts with cytosolic ZNF268a to catalyze the phosphorylation of Serine 178 of ZNF268a, which prevents the degradation of ZNF268a, resulting in the stabilization and accumulation of ZNF268a in the cytoplasm. In turn, stabilized ZNF268a recruits the lysine methyltransferase SETD4 to TBK1. SETD4 induced the mono-methylation of TBK1 on lysine 607, which is critical for the activation of TBK1. Deficiency of either ZNF268a or SETD4 disrupts the TBK1 signaling complex, thus damaging virus-induced innate immune response signaling (Fig. 9). Together, we conclude that virus infection triggers TBK1-mediated ZNF268a phosphorylation, which subsequently leads to ZNF268a stabilization and recruitment of SETD4 to TBK1, followed by TBK1 mono-methylation, thereby potentiating TBK1 for antiviral immune signaling.

In Figure 2*G*, a partial but not complete rescue effect was possibly caused by insufficient expression of ZNF268a construct, as ZNF268a was extremely difficult to overexpress by transient transfection, as we had mentioned in our previous work (31). Similar examples of poor expression efficiency could also be found in a murine KRAB zinc finger protein ZFP809 (32, 33). The technical difficulty impeded our investigation of ZNF268a′s biological function. However, this tightly restricted expression pattern may also imply that aberrantly high expression of ZNF268a could be toxic to cells; therefore, it could be targeted by the selective autophagic receptor Tollip and be kept at a low level under physiological conditions. In contrast, under stressed conditions like viral infection, the ZNF268a expression level has to be temporally and rapidly elevated to facilitate antiviral immunity, demonstrating a subtle balance between the low and high expression patterns of ZNF268a.

Prior work in our laboratory has shown that the ZNF268 gene encodes at least two protein isoforms: the full-length ZNF268a and the truncated ZNF268b2 (38). ZNF268a is a canonical KRAB-zinc finger protein with a KRAB domain at the N-terminal and an array of 24 C2H2 zinc finger motifs at the C-terminal. ZNF268b2 lacks the N-terminal KRAB domain and only retains 24 C2H2 zinc fingers along with a spacer domain linked to the KRAB domain (30, 38, 39). In terms of the functions of ZNF268, our previous work on ZNF268a (31) and ZNF268b2 (40) suggests that ZNF268a/b2 both bind the IKK complex, which is a crucial component of NF-κB signaling cascade reasonably equivalent to the role of TBK1 in IRF3 signaling. In our prior study, we showed that the zinc finger arrays are responsible for mediating IKKα association (31). How ZNF268a interacts with TBK1 is still undetermined. We speculate that the long zinc finger array could act as a large scaffold to recruit TBK1, similar to IKKα binding. ZNF268a consists of 24 zinc fingers, there could be a grammar code for the binding specificity of ZNF268a. For example, ZNF268a may utilize different sets of zinc finger combinations to bind IKKα and TBK1, respectively. Future investigation should focus on identifying the minimally essential zinc finger motif required for IKKα/TBK1 association. ZNF268b2 harbors all the zinc fingers as the full-length ZNF268a. Whether ZNF268b2 is involved in antiviral immune regulation and if so, what could be the role also remain to be answered. A more detailed comparison is needed to elucidate whether a conceptual framework exists to understand the functions of ZNF268a and ZNF268b2 in other biological signaling pathways.

Of note, accumulating evidence indicates that non-histone protein lysine methylation emerges as a novel mechanism to regulate various physiological and pathological processes (41). In tumorigenesis, lysine methyltransferase-mediated p53 methylation exerts distinct effects in cancer cells (42, 43). Furthermore, lysine methyltransferase SETD6 has been reported to suppress RelA/p65 activation *via* K310 mono-methylation (44). Additionally, methyltransferase SETD2-mediated STAT1 is critical for interferon antiviral activity (45). SETD4 is reported to epigenetically control breast quiescent cancer stem cells (46, 47). Setd4 also regulates the quiescence of c-Kit+ cells in the adult mouse heart (48) and contributes to pancreatic development (49). Besides, SETD4 is shown to regulate cell quiescence during diapause formation in Artemia (36). Interestingly, a recent paper reports that SETD4 positively regulates TLR agonist-induced expression of NF-κB-dependent cytokines in mouse BMDMs (50). The underlying molecular mechanism involves epigenetically catalyzing the trimethylation of H4K20. It is still unknown whether SETD4 could function as a non-histone methyltransferase. In this study, we provide experimental evidence that SETD4 catalyzes the mono-methylation of TBK1, expanding our understanding of SETD4 as a novel non-histone methyltransferase. During our manuscript preparation, Yan *et al*. (22) published a paper reporting that the methyltransferase PRMT1 could catalyze methylation of TBK1 on six key arginine residues and enhance TBK1 activation by promoting TBK1 aggregation. Along with their work, our current study would further add more insights into the importance of methylation regulation in the innate immune response.

Another interesting point is that ZNF268a serine 178 and TBK1 lysine 607 are both conserved across primates but absent or variable in rodents (Fig. S5, B and C), which implies a possible species-specific feedback loop in the regulation of innate immunity signaling. Therefore, we hypothesize that ZNF268a-mediated TBK1 activation and TBK1-mediated ZNF268a stabilization could be a unique host defense mechanism in primate evolution. On the other hand, we tried to reconstitute rodent TBK1 in human cells and examined the effects of virus-induced interferon response. Though mTBK1 displayed impaired activation of IFN-β reporter and IFNB expression, we did not observe a substantial increase of IFN-β reporter activation and IFNB expression in mTBK1 R607K mutant rescued HEK293T TBK1−/− cells (data not shown), which may indicate additional species-specific factors involving the regulation of TBK1 activation.

In summary, our study reveals a novel mechanism by which ZNF268a phosphorylation and TBK1 methylation act cooperatively to regulate innate immunity and provides insights for understanding innate immune signaling transduction.

## Materials and methods

### Cells and viruses

Human embryonic kidney (293T) cells and human monocytic (THP-1) cells were cultured in Dulbecco’s modified Eagle’s medium and Roswell Park Memorial Institute (RPMI) 1640 medium, both supplemented with 10% fetal bovine serum and 1% (w/v) each of penicillin and streptomycin.

In the mentioned experiments, THP1 cells were differentiated before stimulation using 100 nM phorbol 12-myristate 13-acetate (PMA) for 24 h and then rested in a medium lacking PMA for 24 h.

HSV-1 was a gift from Dr Shuwen Wu of the College of Life Science, Wuhan University.

### Nucleic acid transfection and virus infection of cultured cells

Hieff Trans Liposomal Transfection Reagent was used for transfection of plasmid DNAs and poly(I:C) (Invivogen) according to the manufacturer’s instructions.

Infection of SeV, VSV, and HSV-1 was performed as described in (51). Briefly, viruses with 0.5 to 5 M.O.I were added into the fresh and serum-free medium, and cells were incubated at 37 °C in 5% CO_2_ (v/v) for 1 h, shaking mildly every 15 min. Virus-containing medium was then replaced by a fresh medium containing 10% FBS.

### Expression plasmids and reagents

The plasmids used in this study were described in a previous study (31). Mutant constructs for TBK1 (TBK1-K607A, TBK1-K607R and TBK1-K584A) and ZNF268a (ZNF268a-S178A, ZNF268a-S178D) were generated using WT TBK1 and ZNF268a cDNA clone as templates *via* the KOD-Plus-Mutagenesis kit (Toyobo).

### Luciferase reporter gene assay

HEK293T cells were seeded into 24-well plates (1 × 10^5^ cells/well). After 24 h, the cells were transfected with the indicated plasmids (400 ng), the IFNB or ISRE reporter plasmid (100 ng), and the Renilla luciferase plasmid (5 ng) as control. After transfection for 24 h, the cells were infected with SeV for 12 h, and luciferase activity was measured with the Dual-Luciferase Reporter assay kit (Promega Corporation). We normalized the data by calculating the ratio between firefly luciferase activity and Renilla luciferase activity.

### RNA-mediated interference

The Lonza Amaxa Nucleofector kit was used to transfect THP-1 cells with siRNAs (30 pmol/sample) using an optimized Nucleofector Program U-001. For siRNA transfection in HEK293T cells, we used Lipofectamine 2000 (Invitrogen) according to the manufacturer’s instructions. The siRNA sequences were listed as follows: siNC: 5′- UUCUCCGAACGUGUCACGU-3′; siZNF268a-1: 5′- GGAGUGUGAUGUUGGAGAA-3′; siZNF268a-2: 5′- GCAGAAGAGUCGCAGAAUA-3′; siZNF268a-3: 5′- GGAAAACUAUGUCUUCUUA-3′; siSETD4-1: 5′- GGAAGUUUCAAGAUUCAAA-3′; siSETD4-2: 5′- GGAUCAUGGCUAUAUAGAA-3′; siSETD4-3: 5′- CAAGAGAAAUACUUGUUAA-3′.

### Subcellular fractionation

Cells were homogenized in hypotonic buffer (10 mM HEPES [pH 7.9], 1.5 mM MgCl_2_, 10 mM KCl) supplemented with protease inhibitors (MCE) by 20 passages through a 1-mL syringe, followed by centrifugation at 1500*g* for 5 min at 4 °C. The supernatant contained the crude cytoplasmic fraction. The protein concentration of the cytoplasmic fractions was measured by BCA, and the cytoplasmic fraction was then subjected to immunoblot analysis. For the assay in Figure 2*F*, the post-cytoplasmic pellets were washed three times with hypotonic buffer and lysed with high-salt lysis buffer (20 mM HEPES [pH 7.9], 1.5 mM MgCl_2_, 1.4 M NaCl, 0.2 mM EDTA, 25% glycerol) plus protease inhibitors (MCE). After sonication and centrifugation at 12,000*g* for 10 min at 4 °C, the supernatant contained the nuclear fraction and would be used for immunoblotting.

### Protein fractionation in sucrose gradient

Cells were lysed in a hypotonic buffer by repeated douncing. After differential centrifugation, as described above, cytoplasmic fractions were further purified by sucrose density ultracentrifugation. In brief, cytoplasmic fractions were loaded on top of a centrifuge tube containing 1 ml of 60% sucrose in PBS on the bottom layer and 1 ml of 10% sucrose in PBS on the top layer, with 1ml of 30% and 20% sucrose in PBS laid in between. After centrifugation at 160,000*g* for 2 h, fractions with equal volume were taken from the top to bottom of the tube and analyzed by immunoblot.

### Immunoprecipitation and immunoblot analysis

Cells were lysed with lysis buffer (20 mM Tris-HCl [pH7.4], 150 mM NaCl, 10% glycerol, 1% NP-40) containing protease inhibitors (MCE) and phosphatase inhibitors (MCE) for 30 min on ice. After centrifugation at 12,000*g* for 15 min, the protein concentrations of the lysates were measured by BCA assay (Thermo Fisher Scientific). Immunoblot analysis was performed using 10 to 30 μg samples of the lysates. For immunoprecipitation, equal amounts of the cell lysates were incubated with Dynabeads Protein G conjugated with specific antibodies at 4 °C overnight. The next day, the precipitants were washed four times with lysis buffer, and the immunocomplexes were eluted with sample buffer containing 1 × SDS loading buffer for 10 min at 95 °C. The immunoprecipitated proteins were then separated by SDS-PAGE.

The antibodies used for immunoblot analysis, immunoprecipitation, and immunofluorescence were as follows: Anti-DDDDK-tag mAb (Clone: FLA-1), Anti-HA-tag mAb (Clone: TANA2) were from MBL; HA tag Rabbit Polyclonal antibody (51064-2-AP), IRF3 Polyclonal Antibody (11312-1-AP), GST Tag Polyclonal Antibody (10000-0-AP) were from Proteintech; Anti-NAK/TBK1 (ab109735), Anti-NAK/TBK1 (phosphor-S172) antibody (ab109272) were from Abcam; Phospho-IRF-3 (Ser396) (D6O1M) Rabbit mAb (29047) was from Cell Signaling Technology; The affinity-purified antibody of ZNF268a was customized and prepared by Genscript, and the antibody of ZNF268b2 was homemade as described in previous study (40). Species-specific HRP-conjugated IgGs were used as secondary antibodies except for Figure 2, *B*, *D* and *F*, in which Dylight 680 and Dylight 800-conjugated secondary antibodies were used.

### Native PAGE

HEK 293T cells were harvested with 50 μl ice-cold lysis buffer (20 mM Tris-HCl, pH 8.0; 137 mM NaCl; and 0.5% NP-40 containing protease inhibitor cocktail). After centrifugation at 12,000 rpm for 10 min, the supernatant protein was quantified and diluted with 2 × native PAGE sample buffer (125 mM Tris-HCl, pH 6.8; 30% glycerol; and 0.1% bromophenol blue) and then 30 μg total protein was immediately applied to a pre-run 7.5% native gel for separation. After electrophoresis, proteins were transferred onto a PVDF membrane for immunoblotting.

### GST-protein purification

GST-ZNF268a N terminus and GST-SETD4 SET full-length and mutants were expressed from *E. coli* Rosetta 2 (DE3) cells and purified under native conditions. Briefly, *E. coli* were grown to OD600 of 0.6 and induced with 1 mM IPTG at 28 °C overnight. Pelleted cells were resuspended in lysis buffer (50 mM Tris-HCl pH 7.5, 150 mM NaCl, 1 mM DTT, protease inhibitor). After sonication, lysates were pelleted at 30,000*g* at 4 °C for 20 min. Supernatants were applied to GST purification with 1 ml ProteinIso GST Resin (Transgen) prewashed with lysis buffer at 4 °C. Proteins were eluted with 10 mM glutathione (Sigma) in lysis buffer. The eluted protein can be dialyzed against a buffer of choice at 4 °C using the ultrafiltration centrifuge tube.

### Enzyme-linked immunosorbent assay

Supernatants from stimulated cells were harvested 24 h after stimulation. The concentrations of IFN-β in culture supernatants were measured using the VeriKine Human Interferon Beta ELISA Kit from PBL according to the manufacturer’s instructions.

### RNA isolation and RT-qPCR

Cells were harvested in TRIzol as per the manufacturer’s instructions. Complementary DNA (cDNA) was generated using Hifair II first Strand cDNA Synthesis SuperMix for qPCR (gDNA digester plus). Real-time PCR was conducted with SYBR Green (Yeasen) on an Applied Biosystems ABI7500 real-time PCR system (Thermo Fisher Scientific) following standard protocols. PCR primer sequences are listed in the Supplementary Materials.

### Ubiquitination detection

For analysis of the ubiquitination of TBK1 in ZNF268a knock-out and wild-type HEK293T cells, cells were transfected with Flag-TBK1, HA-ubiquitin mutants (K63 only), then the cells were infected with SeV for 8 h, whole-cell extracts were prepared under denatured conditions using RIPA buffer containing 1% SDS, followed by diluted with lysis buffer to reduced SDS concentration to 0.1% and immunoprecipitated with anti-Flag and analyzed by immunoblot with anti-HA.

### Immunofluorescence and confocal microscopy

Cultured cells were fixed with 4% (w/v) paraformaldehyde for 30 min at 4 °C and permeabilized with 0.5% (v/v) Triton X-100 containing 1% BSA for 30 min at room temperature. Subsequently, the cells were incubated with primary antibody in buffer containing 1% (w/v) BSA and 0.05% (v/v) Triton X-100 overnight, followed by incubation with Alexa Fluor 488/594-conjugated secondary antibody (Thermo Fisher Scientific) for 1 h at 37 °C. The cells were then washed three times, and the nuclei were stained with DAPI. Images were acquired using Leica TCS SP8. Colocalization analysis is performed by Fiji Coloc two and Pearson’s coefficient is calculated using the Bisection threshold regression method.

### CRISPR-Cas9-mediated genome editing

ZNF268a KO HEK293T cells were established as described in a previous study (31). We cloned sgRNAs into the pGL3-U6-sgRNA-PGK-puromycin vector (52) and co-transfected the constructs along with pST1374-NLS-flag-linker-Cas9 into HEK293T cells. After allowing 24 h for transfection, we placed the cells under puromycin (1 μg/ml) and blasticidin (3 μg/ml) selection for a minimum of 3 days. We then picked single clones, cultured them, and identified them by sequencing. gRNA sequences are listed in the Supplementary Materials.

### *In vitro* kinase assay

For the *in vitro* phosphorylation assay, full-length active TBK1 protein was purchased from Abcam. The WT and S178A GST-ZNF268a N terminus fusion proteins were mixed at the indicated amount with TBK1 in a 0.2 ml tube. The kinase reactions were performed in the buffer 20 mM HEPES-KOH pH 7.0, 2 mM ATP, 5 mM MgCl_2_ and protease inhibitor cocktail. The reaction was incubated at 30 °C for 1 h and then resolved by SDS-PAGE followed by Western blot or mass spectrometry analysis.

*In vitro* methylation assays were performed by combining 1 mg of recombinant GST-SETD4 SET fusion protein or 4 × 150mm culture dishes of HEK293T cells expressed Flag-SETD4 with 0.2 mg recombinant TBK1 in a methyltransferase buffer (50 mM Tris pH 8.0, 20 mM KCl, 5 mM MgCl_2_, and 10% glycerol) supplemented with 1 mM S-adenosyl-methionine (SAM, Sigma-Aldirich). The reaction mixtures were incubated overnight at 30 °C. Reactions were resolved by SDS-PAGE, followed by immunoblot or mass spectrometry analysis.

### Nano-liquid chromatography/tandem MS (nano LC-MS/MS) analysis

Nano-LC/tandem MS analysis for protein identification and label-free quantification was performed by the College of Life Science Core services of Wuhan University. Briefly, tryptic peptides were separated on a C18 column and analyzed by Q Exactive HF (Thermo). Proteins were identified using the search engine of the National Center for Biotechnology Information against the human RefSeq protein databases.

### Quantification and statistical analysis

Statistical significance between groups was determined by unpaired two-tailed Student’s t-tests performed using GraphPad Prism 8.0. Differences were considered significant when *p* < 0.05 (∗*p* < 0.05, ∗∗*p* < 0.01, ∗∗∗*p* < 0.001, ∗∗∗∗*p* < 0.0001).

## Data availability

The cytoplasmic ZNF268a-interacting proteome of HEK293T upon SeV infection, TBK1-mediated ZNF268a phosphorylation site identification and SETD4-mediated TBK1 methylation site identification MS data from this publication have been deposited to the ProteomeXchange Consortium (http://proteomecentral.proteomexchange.org) *via* the iProX partner repository with the dataset identifier PXD041079, PXD041080 and PXD041084, respectively.

All MS data are accessible *via* iProX for links as below:

PXD041079: https://www.iprox.cn//page/project.html?id=IPX0006153000;

PXD041080: https://www.iprox.cn/page/project.html?id=IPX0006155000;

PXD041084: https://www.iprox.cn/page/project.html?id=IPX0006156000.

## Supporting information

This article contains Supporting information.

## Conflicts of interests

The authors declare that they have no known competing financial interests or personal relationships that could have appeared to influence the work reported in this paper.

## Figures and Tables

**Supplemental Fig. S3:**
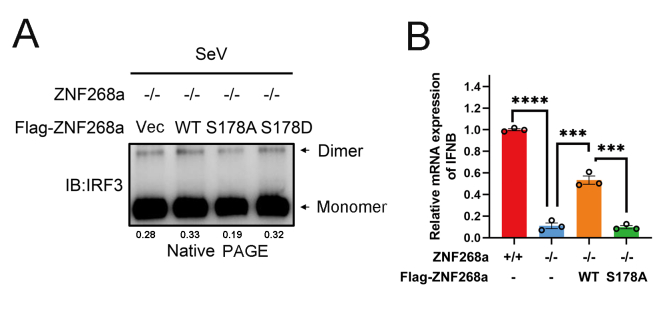
**Related to**Figure 5**ZNF268a S178 phosphorylation deficiency failed to restore virus-induced interferon signaling.***A*, Flag-ZNF268a WT, S178D and S178A constructs were re-introduced into ZNF268a KO HEK293T cells; after SeV infection for 8 h, IRF3 dimerization was detected by native PAGE and immunoblot. *B*, qPCR analysis of IFNB transcription of ZNF268a KO HEK293T cells transfected with Flag-ZNF268a WT and S178A, infected with SeV for 12 h. Values are expressed as the mean ± SEM and unpaired two-tailed Student’s t-tests are used for statistical analysis in which ∗*p* < 0.05, ∗∗*p* < 0.01, ∗∗∗*p* < 0.001, ∗∗∗∗*p* < 0.0001. Data are representative of three independent experiments.
